# Exaggerated blood pressure response to static exercise in hindlimb ischemia-reperfusion

**DOI:** 10.3389/fphys.2022.1048559

**Published:** 2022-12-14

**Authors:** Lu Qin, Jianhua Li

**Affiliations:** Heart and Vascular Institute, The Pennsylvania State University College of Medicine, Hershey, PA, United States

**Keywords:** peripheral artery disease, muscle afferent, hindlimb ischemia-reperfusion, blood pressure, sympathetic nerve activity

## Abstract

Peripheral artery disease (PAD) reduces the blood flow supply in the affected limbs as one of the significant cardiovascular concerns. Revascularization surgery in the femoral artery plays a central role in treating PAD. Exercise is also a rehabilitation strategy suggested for PAD patients to improve vascular functions. However, the effects of limb ischemia-reperfusion (IR), one of the most predominant complications in revascularization surgery, on exercise-induced arterial blood pressure (BP) response are poorly understood. In the present study, we determined 1) the blood flow status in the hindlimb muscles of rats (plantar muscle, red and white portions of gastrocnemius) with different time points of the hindlimb IR; and 2) the BP response to static muscle contraction in rats at different time points after the blood flow reperfusion procedure. Results of this study indicated that, compared with the Sham group, the blood flow in the hindlimb muscles evaluated by Evans blue concentration was significantly reduced at 6 h of femoral artery occlusion (FAO 6 h) (vs. sham control, *p* < 0.05). The decreased blood flow was gradually recovered after the blood flow reperfusion for 18 (IR 18 h), 66 (IR 66 h), and 114 (IR 114 h) hours (*p* < 0.05 vs. FAO 6 h for all IR groups). The response of mean arterial pressure was 20 ± 4 mmHg in Sham rats (*n* = 7); 32 ± 10 mmHg in IR 18 h rats (*n* = 10); 27 ± 7 mmHg in IR 66 h rats (*n* = 13); 26 ± 4 mmHg in IR 114 h rats (*n* = 9) (*p* < 0.05 vs. Sham for all groups). No significant difference was observed in the peak-developed tension during muscle contraction among all the groups (*p* > 0.05). In conclusion, static exercise-induced BP response is exaggerated following IR. Whereas the BP response is not statistically significant but tends to decrease with a prolonged IR time, the exaggerated BP response remains through time points from post-IR 18 h–114 h.

## Introduction

Peripheral artery disease (PAD) is one of the significant cardiovascular concerns. It affects approximately 200 million population worldwide who are older than 40 years old, and the prevalence rate of the disease dramatically increases from the age group of 70–79 years old (Virani et al., 2020). Due to atherosclerotic vascular disease, progressive narrowing of the lower extremity leads to severe limb ischemia. This pathophysiological change essentially limits walking tolerance and jeopardizes the daily mobility performance in PAD patients (McDermott et al., 2004).

Similar to the significant advances seen in the management of other cardiovascular diseases, such as the by-pass surgery for coronary artery disease, the surgery means of revascularization is one of the most effective treatment strategies to restore the blood flow of the affected limb and plays a central role in the therapeutic management of PAD (Vartanian & Conte, 2015). Meanwhile, the supervised exercise intervention has been proposed as one of the most effective rehabilitation means for PAD patients in both non-surgery and post-surgery situations (Hamburg & Balady, 2011; Fakhry et al., 2015). However, it is essential to note that the surgical process of revascularization brings the blood flow back to the ischemic tissues but inevitably induces ischemia-reperfusion (IR) injury to the re-perfused tissues, e.g., skeletal muscle, vasculature, and innerved sensory nerves (Kalogeris, Baines, Krenz, & Korthuis, 2012). Non-etheless, the mechanisms by which IR affects exercise ability in PAD patients following revascularization are still poorly understood.

**FIGURE 1:**
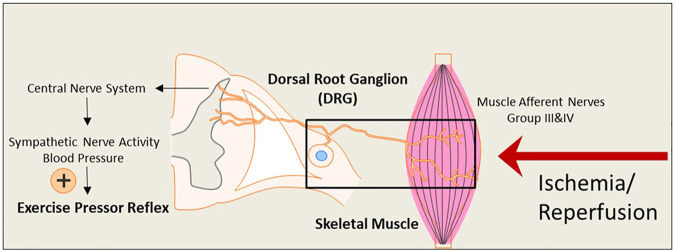
An illustrative diagram of the exercise pressor reflex (EPR) and the hypothesized impact of the ischemia-reperfusion on the neural pathway leading to the reflex. During exercise, the EPR is initiated as thin fiber afferents arising from contracting skeletal muscle. The signals sensed by the nerve terminals embedded in the skeletal muscle are transmitted through the primary sensory neurons of dorsal root ganglion (DRG) to cardiovascular nuclei in the brain stem. The sympathetic nerve activity (SNA) and blood pressure (BP) are therefore increased. Under ischemia-reperfusion conditions, metabolites are elevated in the affected skeletal muscle. This is likely to amplify the receptors in muscle afferent nerves responsive to muscle metabolites and exaggerate the EPR.

During exercise, sympathetic nerve activity (SNA) increases, and this leads to rises in arterial blood pressure (BP) and heart rate (HR), myocardial contractility, and peripheral vasoconstriction due to two mechanisms: central command (Freyschuss, 1970; Goodwin, McCloskey, & Mitchell, 1972; Eldridge, Millhorn, Kiley, & Waldrop, 1985; Vissing, Scherrer, & Victor, 1991; Victor, Secher, Lyson, & Mitchell, 1995) and the “Exercise Pressor Reflex” (EPR) (Coote, Hilton, & Perez-Gonzalez, 1971; McCloskey & Mitchell, 1972; Mitchell, Kaufman, & Iwamoto, 1983; Victor, Bertocci, Pryor, & Nunnally, 1988; Sinoway et al., 1989; Kaufman & Forster, 1996). As illustrated in Figure 1, the EPR is initiated as thin fiber afferents arising from contracting skeletal muscle are engaged (McCloskey & Mitchell, 1972; Mitchell et al., 1983; Kaufman & Forster, 1996). This system responds to mechanical deformation of the muscle afferents receptive field (mechano-receptor) as well as to muscle metabolic by-products (metabo-receptor) (Kaufman & Forster, 1996; M. P. Kaufman, Rybicki, Waldrop, & Ordway, 1984; Rotto & Kaufman, 1988). It has been commonly accepted that the EPR is exaggerated in PAD (Ritti-Dias et al., 2011). This reflex mechanism is explained for a greater BP rise in PAD patients during walking than in healthy control subjects (Baccelli et al., 1999; Bakke, Hisdal, Jorgensen, Kroese, & Stranden, 2007). The augmented BP response affects exercise tolerance and is also closely associated with a higher incidence of cardiovascular events (Lewis et al., 2008). Thus, studying the EPR and regulatory signaling pathways in PAD which experiences intermittent ischemia and reperfusion, is essential.

In a foundational study, the dynamic exercise-induced BP response was first reported to be augmented in a mice model with 6 h of ischemia and 18 h of reperfusion in the forelimb brachial artery (Ross, Queme, Shank, Hudgins, & Jankowski, 2014). However, PAD is more precisely defined as “lower extremity PAD” and specifically referred to as atherosclerotic obstruction from the aortoiliac segments to the pedal arteries (Criqui et al., 2021). In addition, dynamic exercise is a global stimulation of the cardiovascular system. Non-etheless, approaches used in the current study, including static muscle contraction induced by the lumbar ventral root stimulation, are more effective and specifically demonstrative for the EPR response during activation of the muscle metabo- and mechano-sensitive afferent nerves. Considering that the abnormalities in BP regulation in PAD, especially the underlying mechanisms and regulatory factors leading to the exaggerated EPR, have still been poorly understood. This continuously existing exaggeration of BP condition following blood flow reperfusion raises safety concerns for adhering to the exercise intervention strategies during the post-surgery time of PAD. In addition, the time course investigation for the blood flow and the effect of IR on the BP response during exercise is an essential foundation work to study the underlying mechanisms. On top of this, results of the current study would provide potential information determining an optimal time point for the treatment intervention. Yet, to date, to the best of our knowledge any study in those regards has not been conducted.

Accordingly, in the present study, we firstly utilized Evans blue evaluation to validate the blood flow status at different time points during the ischemia-reperfusion stages (control baseline, 6 h of femoral artery occlusion, 18, 66, and 114 h of the blood flow reperfusion). The time courses for this surgical approach were employed to closely mimic an acute IR condition in the lower extremity in human PAD patients. Secondly, we performed experiments in decerebrated, unanesthetized rats to evaluate the BP response following static muscle contraction. Groups of animals included sham rats (sham) and rats with 6 h of hindlimb ischemia following three reperfusion time lengths: 18 h (IR 18 h), 66 h (IR 66 h) and 114 h (IR 114 h). It was hypothesized that the pathophysiological process of IR induces an increase in BP response during static muscle contraction. In contrast, reperfusion surgery can reverse the reduced blood flow in the skeletal muscle of the ischemic hindlimb.

## Methods

### Ethical approval

The experimental animal procedures were performed in compliance with the National Institutes of Health guidelines. They were approved by *the Institutional Animal Care and Use Committee* of the Pennsylvania State University College of Medicine. Male Sprague-Dawley rats (200–300 g) were obtained from Charles River Laboratory and housed in individual cages with free access to food and water. They were kept in a temperature-controlled room (25°C) on a 12-h/12-h light/dark cycle.

### Experimental animals and categorization

The hindlimb IR and sham surgery procedures were performed in different groups of rats. Those with the surgery of femoral artery occlusion (FAO) served as “FAO rats,” while those with IR surgery served as “IR rats”. Specifically, FAO rats that underwent 6 h of FAO-induced hindlimb ischemia were referred to as the “FAO 6 h” group. IR rats were divided into IR 18h, IR 66h, and IR 114 h groups based on the time length of the reperfusion (18, 66, and 114 h, respectively). Those with sham surgeries served as “sham rats”.

### Ischemia-reperfusion procedure

To perform the surgery of the hindlimb IR, rats were anesthetized by inhalation of 2–5% isoflurane in 100% oxygen. A ligature was placed tightly with a slipknot around the femoral artery ∼3 mm distal to the inguinal ligament. After the surgical wound was closed, the rats were returned to the cage and maintained in the lab room. Six hours later, rats were re-anesthetized by inhalation of 2–5% isoflurane in 100% oxygen. Reperfusion was induced by releasing the slipknot to return blood flow into the femoral artery. Surgical forceps were used to gently massage the artery to remove the blood clot and recover the blood flow. After observing the blood flow entering the previously ligated femoral artery, the surgical wound was carefully closed. The same procedures were followed to obtain sham-controls except that a suture was placed below, but the artery was not tied. Buprenorphine hydrochloride (0.05 mg/kg, subcutaneously) was administered for postoperative pain relief before the surgery. Following the surgery, the animals were kept in the surgery room for 2–3 h for observation, and then returned to the animal facility before experiments were performed.

### Evans blue experiment

**FIGURE 2:**
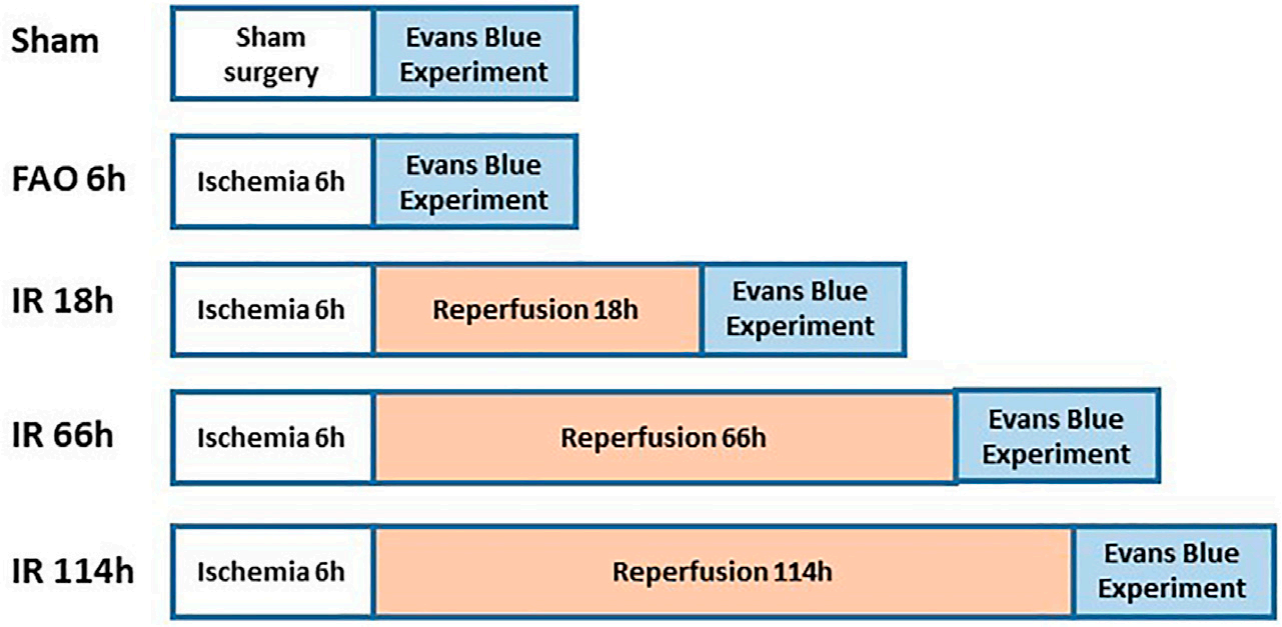
Animal grouping and study protocols in Evans blue experiment.

Figure 2 shows the experimental design on groups of animals during Evans blue study. The previous studies referred to the protocol of the Evans blue injection (Seo et al., 2008; Wang & Lai, 2014). In brief, the left or right jugular veins were exposed, and the distal end was ligated. A small incision was made on the proximal end to the ligation site, and a catheter (PE10) was inserted into the external jugular vein from the incision towards the proximal end. One ml of Evans blue (40 mg/ml in 0.9% saline) solution was injected manually into the external jugular vein catheter, with an injection duration of ∼30s (controlled by using a timer), in animals of experimental groups, followed by a small volume of heparinized saline to flush the line. Then, the plantar muscles from the hind paws were collected in 1 min after the end of the injection; the red portion in the deep part of the lateral head and the white portion in the superficial part of the medial head of gastrocnemius muscle (Winder, Baldwin, & Holloszy, 1974; Matsakas et al., 2006) collected in 20 min, after the end of Evans blue injection. We examined the Evans blue concentration in those above specific parts as the blood flow in the plantar muscle represents the blood flow in the hind paw, and the gastrocnemius muscle is one of the major muscles involved in the hindlimb movement as well as the contracting muscle during evoking of static-muscle-contraction-induced EPR observed in the whole animal study. Collected samples (about 70 mg) were immersed in 1 ml of formamide and incubated at ∼60°C for 24 h. Following the incubation, the samples were brought to room temperature and then were centrifuged for 30 s at 9,000 rpm at room temperature. The supernatant (200 µl) was collected and added to a 96-well plate (Fisher Scientific). Along with the standard curve with Evans blue concentration as 0, 0.15625, 0.3125, 0.625, 1.25, 2.5, 5.0, and 10 μg/ml, the absorbance of the supernatant was measured at 620 nm by a microplate reader. The concentration of Evans blue was then calculated as μg/g tissue.

### Examination of the BP response

**FIGURE 3:**
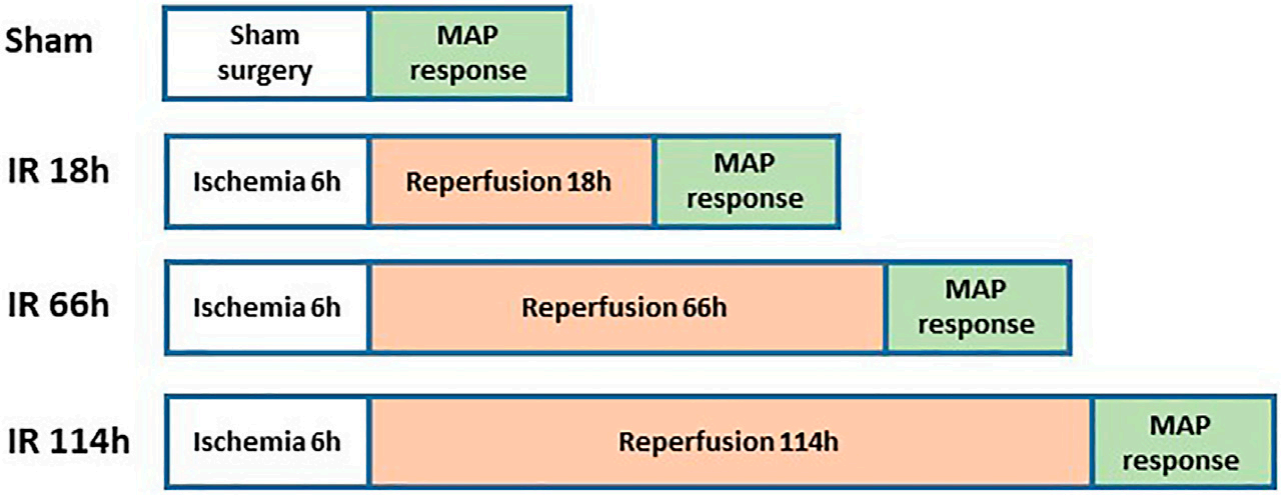
Animal grouping and study protocols for evaluation of BP response.

Figure 3 shows the experimental design on animal groups for evaluating BP response. The rats were anesthetized with a mixture of 2–5% isoflurane and oxygen and ventilated as described previously (Xing, Lu, & Li, 2018). The jugular vein and common carotid artery were cannulated. Fluids were delivered *via* the jugular vein while a pressure transducer was connected to the common carotid artery to measure arterial BP. HR was determined from the arterial pressure pulse.

Decerebration was performed to eliminate the effects of anesthesia on the reflex pressor response. Before the procedure, dexamethasone (0.2 mg, i. v.) was injected to minimize brain stem edema. After decerebration, anesthesia was withdrawn from the rats, and the animals were switched to a ventilator. Then experiments were performed 60 min later.

A laminectomy procedure was performed to expose the spinal cord’s lower lumbar and upper sacral portions. The peripheral ends of the transected L4 and L5 ventral roots were placed on platinum bipolar stimulating electrodes (S. A. Smith, Mitchell, & Garry, 2001). Static muscle contractions were induced by electrical stimulation of the L4 and L5 ventral roots (30s, 3× motor threshold with a duration of 0.1 ms at 40 Hz). The reflex BP during muscle contraction was recorded in sham and IR rats. At the end of the experiments, the animals were euthanized by inhalation of an overdose of isoflurane followed by cardiac puncture.

### Statistical analysis

Unless specified, the data in this study were presented as the mean ± standard deviation (SD). The SPSS for Windows version 26.0 was utilized for all statistical analyses. Kolmogorov-Smirnov test was applied to evaluate the normality, and Leven’s test was used to assess the equality of the variance. Once the data met the standard of normal distribution and equal variance, one-way ANOVA was applied to compare the differences in BP and HR responses among the groups (Sham, IR 18 h, IR 66 h, and IR 114 h). As appropriate, Tukey’s *post hoc* test was applied to compare the differences between specific groups. If the data did not meet the standard of normal distribution and equal variance, e.g., data on the MAP response, baseline HR and Evans blue concentration in the white portion of the gastrocnemius muscle, non-parametric Kruskal–Wallis test, and Mann-Whitney test were applied. A *p*-value less than 0.05 was considered statistical significance.

## Results

### Evans blue concentration in plantar and gastrocnemius muscle

**FIGURE 4:**
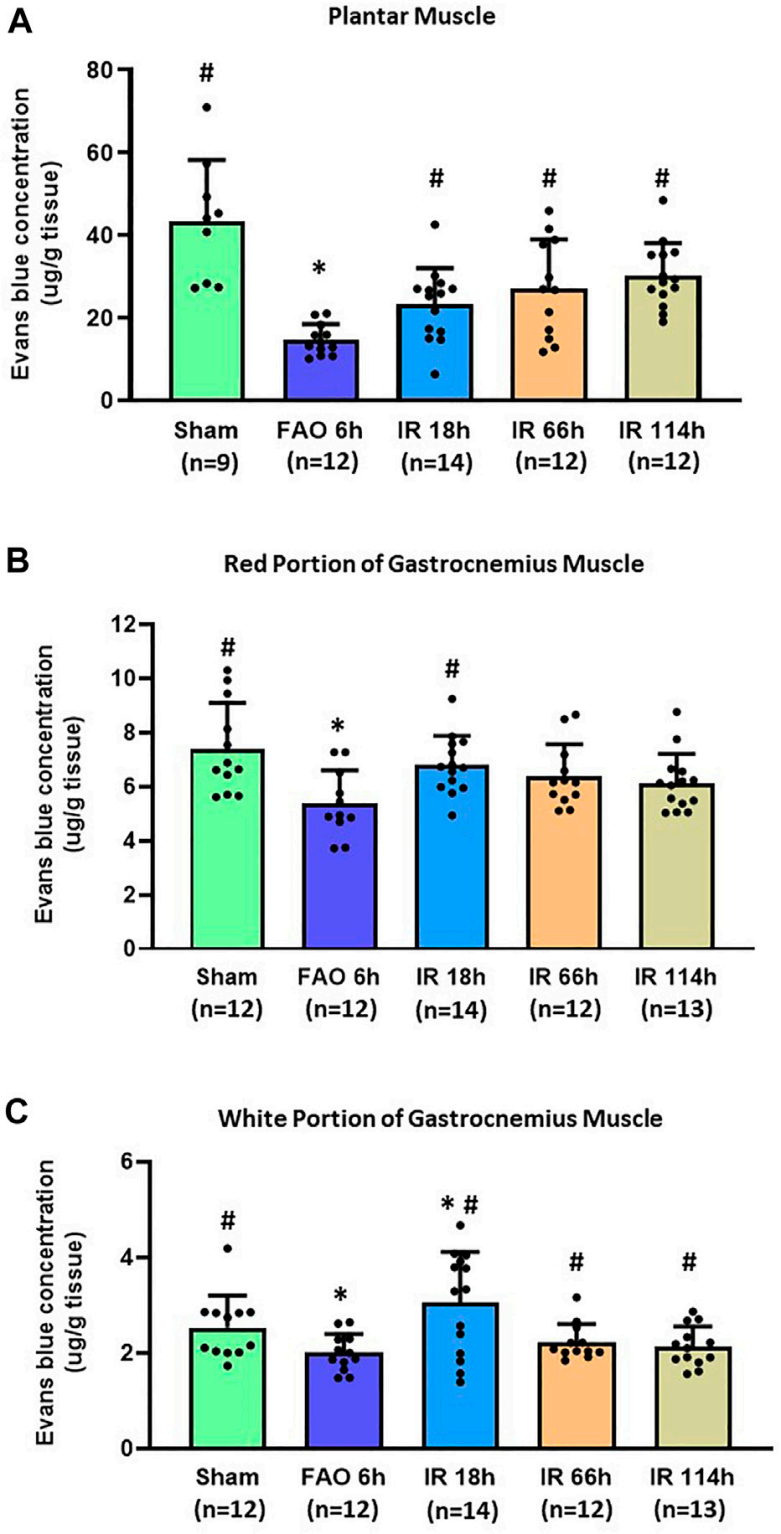
Evans blue concentration in the hindlimb plantar muscle and gastrocnemius muscle in different time points of IR. Experiments were performed in male Sprague-Dawley rats (200–300 g). Data presented as Mean ± SD **(A)**, Data were analyzed by one-way ANOVA with Tukey’s *post hoc* test. Compared with sham rats, Evans blue concentration was significantly reduced at 6 h after FAO 6 h. It was restored in the three investigated time points following the blood flow reperfusion (IR 18h, IR 66h, and IR 114 h) (all *p* < 0.05) **(B)**, Data were analyzed by one-way ANOVA with Tukey’s *post hoc* test. In the red portion of the gastrocnemius muscle compared with sham controls, Evans blue concentration was significantly reduced at FAO 6h, *p* < 0.05) and restored in IR18 h **(C)**, Data were analyzed by the non-parametric Kruskal–Wallis test and Mann-Whitney test. In the white portion of the gastrocnemius muscle, compared with sham controls, Evans blue concentration was significantly reduced at 6 h after femoral artery occlusion (FAO 6h, *p* < 0.05), and this reduction was recovered to some degree in the three investigated time points following blood flow reperfusion (IR 18h, IR 66h, and IR 114 h) (all *p* < 0.05 vs. FAO 6 h). Sham = sham surgery group; IR = ischemia-reperfusion; FAO 6 h = 6 h’ femoral artery occlusion; IR 18 h = 18 h following blood flow reperfusion; IR 66 h = 66 h following blood flow reperfusion; IR 114 h = 114 h following blood flow reperfusion. *p < 0.05 vs. sham; #*p* < 0.05 vs. FAO 6 h. The number below the bar chart indicates the sample size in each group.

In the sham group, the Evans blue concentration was 43.45 ± 14.78 μg/g tissue in the plantar muscle (*n* = 9), 7.40 ± 1.68 μg/g tissue in the red portion of the gastrocnemius muscle (*n* = 12), and 2.52 ± 0.66 μg/g tissue in the white portion of the gastrocnemius muscle (*n* = 12). Compared with the sham group, FAO 6 h significantly reduced the Evans blue concentration in plantar muscle (14.68 ± 3.76 μg/g tissue, n = 12, vs. sham, *p* < 0.05), in the red portion of the gastrocnemius muscle (5.37 ± 1.23 μg/g tissue, *n* = 11, vs. sham, *p* < 0.05) and the white portion of the gastrocnemius muscle (2.00 ± 0.39 μg/g tissue, *n* = 12, vs. sham, *p* < 0.05) (Figures 4A–C).

After the blood flow reperfusion, the Evans blue concentration increased when compared with 6 h after femoral artery occlusion in plantar muscle (IR 18 h: 23.21 ± 8.79 μg/g tissue, *n* = 14; IR 66 h 27.14 ± 11.83 μg/g tissue, *n* = 14; IR 114 h 30.31 ± 7.84 μg/g tissue, *n* = 12; *p* < 0.05, all groups vs. FAO 6 h), and in the white portion of the gastrocnemius muscle (IR 18 h: 3.04 ± 0.39 μg/g tissue, *n* = 14; IR 66 h 2.22 ± 0.37 μg/g tissue, *n* = 12; IR 114 h 2.13 ± 0.42 μg/g tissue, *n* = 13; *p* < 0.05, all groups vs. FAO 6 h); the Evans blue concentration in IR 18 h was also significantly higher than sham group (*p* < 0.05). In the red portion of the gastrocnemius muscle, the Evans blue concentration in IR 18 h was significantly higher than that in FAO 6 h group (IR 18 h: 6.80 ± 1.07 μg/g tissue, n = 14; *p* < 0.05 vs. FAO 6 h) (Figures 4A–C).

### BP response to static muscle contraction at different time points following IR

**FIGURE 5:**
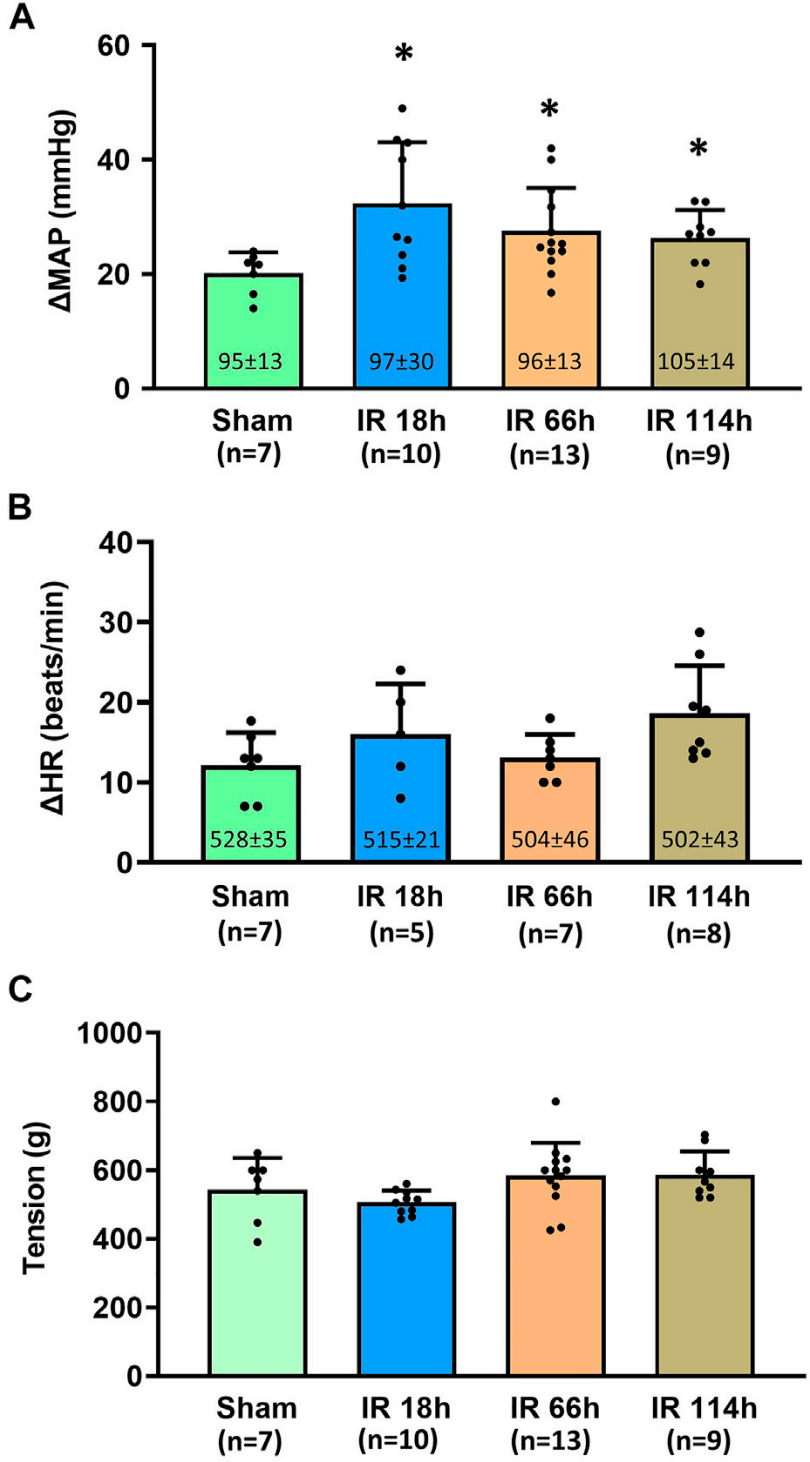
Blood pressure, heart rate response and the muscle development tension in different groups following the static muscle contraction. Experiments were performed in male Sprague-Dawley rats (200–300 g). Data presented as Mean ± SD **(A)**, Data were analyzed by the non-parametric Kruskal–Wallis test and Mann-Whitney test. Compared with sham rats, MAP response was exaggerated in the three investigated time points following IR (all *p* < 0.05). Numbers in parentheses represent baseline values for MAP **(B)**, Data were analyzed by the non-parametric Kruskal–Wallis test and Mann-Whitney test. Compared with sham rats, HR response was not significantly altered in IR 18 h rats, IR 66 h rats and IR 114 h rats (*p* > 0.05) **(C)**, Data were analyzed by one-way ANOVA with Tukey’s *post hoc* test. The development tensions evoked during muscle contraction were similar among the groups (*p* > 0.05). Sham = sham surgery group; IR = ischemia-reperfusion; IR 18 h = 18 h following IR; IR 66 h = 66 h following IR; IR 114 h = 114 h following IR. *p < 0.05 vs. sham. The number below the bar chart indicates the sample size in each group.

Before the development of muscle tension, the baselines of mean arterial pressure (MAP) in four groups of animals are 95 ± 13 mmHg in the Sham group, *n* = 7; 97 ± 30 mmHg, *n* = 10 in IR 18 h group; 96 ± 13 mmHg, *n* = 13 in IR 66 h group; and 105 ± 14 mmHg, *n* = 9 in IR 114 h group (Figure 5A). There was no significant difference among the means of groups (*p* > 0.05).

During the development of muscle tension, the peak MAP was determined for all groups: 1) Sham group: 115 ± 15 mmHg, *n* = 7; 2) IR 18 h group: 130 ± 28 mmHg, n = 10; 3) IR 66 h group: 124 ± 13 mmHg, *n* = 13; and 4) IR 114 h group: 131 ± 14 mmHg, n = 9. The peak MAP response induced by static muscle contraction was calculated as the peak MAP value - baseline MAP value. Compared with the Sham group (MAP response: 20 ± 4 mmHg, *n* = 7), the BP response to static muscle contraction was significantly increased in IR 18 h (MAP response: 32 ± 10 mmHg, n = 13, vs. sham: *p* < 0.05), IR 66 h (MAP response: 27 ± 7 mmHg, n = 13, vs. sham: *p* < 0.05) and IR 114 h (MAP response: 26 ± 4 mmHg, n = 9, vs. sham: *p* < 0.05) (Figure 5A). There was no significant difference observed in the baseline HR (Sham rats: 528 ± 35 beats/min, *n* = 7; IR 18 h rats: 515 ± 21 beats/min, *n* = 5; IR 66 h rats: 504 ± 46 beats/min, *n* = 7; IR 114 h rats: 502 ± 43 beats/min, *n* = 8; *p* > 0.05) and HR responses (Sham rats: 12 ± 4 beats/min, *n* = 7; IR 18 h rats: 16 ± 6 beats/min, *n* = 5; IR 66 h rats: 13 ± 3 beats/min, *n* = 7; IR 114 h rats: 19 ± 6 beats/min, *n* = 8; *p* > 0.05) (Figure 5B). No difference in peak developed tension was seen (Sham rats: 543 ± 92g; IR 18 h rats: 506 ± 35g; IR 66 h rats: 589 ± 72g; IR 114 h rats: 586 ± 94g; *p* > 0.05, Figure 5C) during muscle contraction among all the groups.

## Discussion

The major findings of the present study include, in the rat hindlimb ischemia-reperfusion model: 1) The blood flow in the lower extremity, which is indicated by the Evans blue concentration in the plantar muscle and gastrocnemius muscle, reduces at 6 h after the femoral artery ligation and gradually restores at 18, 66 and 114 h after the blood flow reperfusion in the femoral artery (Figure 4); 2) Compared with the sham group, the BP response to the static muscle contracting of the hindlimb is exaggerated at 18 h after the ischemia-reperfusion (Figure 5A); 3) Whereas the BP response is not statistically significant but tends to decrease with a prolonged IR time, the exaggerated BP response following IR persists at least 114 h after the blood flow reperfusion (Figure 5A).

IR injury is an inevitable complication following revascularization surgery or organ transplants (Arato et al., 2008; Zhai, Petrowsky, Hong, Busuttil, & Kupiec-Weglinski, 2013). To explore the effect of IR on the pain sensory and dynamic exercise-induced BP response, Ross et al. (2014) modified a previously published model (Coderre & Bennett, 2010) by inducing the forelimb ischemia by the bilateral ligation of the brachial artery for 6 h in mice. They examined the effects of IR on group III and IV sensory nerve activity 18 h following the reperfusion. In the present study, we applied ischemia-reperfusion surgery on the rat hindlimb. The reasons for this modification are: 1) PAD is more precisely defined as “lower extremity PAD” and more specifically referred to as atherosclerotic obstruction from the aortoiliac segments to the pedal arteries (Criqui et al., 2021); and 2) The laminectomy procedure on the lower lumber has been performed for electrical stimulation of the ventral roots to evoke static muscle contraction of the hindlimb of rats in a number of our previous studies. Using the same approach in the present study can provide more comparable results evidenced by the exercise-induced BP response generated by static muscle contraction in the hindlimb muscle of rats.

For the length of the ischemic stage before the blood flow reperfusion, we also determined it as 6 h in the present study. This decision was made based on evidence that the tissues (e.g., skeletal muscle, muscle afferent nerve terminals) are under the ischemic condition following the femoral artery occlusion. Of note, in one of our previous studies (Gao & Li, 2013), we evaluated the hypoxia-inducible factor 1α (HIF-1α) expression in the primary sensory nerve-dorsal root ganglion (DRG) in different time courses following femoral artery occlusion. It was found that the HIF-1α in the DRG was significantly enhanced 6 h following the hindlimb ischemia. HIF-1α is an effective biomarker to indicate ischemic conditions of the tissues and involves the regulatory process of the EPR (Qin & Li, 2021). Additionally, previous reports have shown that the ischemia length of 4–6 h induces severe damage in the hindlimb tissues (Belkin, Brown, Wright, LaMorte, & Hobson, 1988). For PAD patients who need revascularization surgery, the blood flow is poorly perfused, and the affected tissues are damaged in the ischemia limb (Kalra et al., 2001; Ortmann et al., 2012). Thus, in the current study, the IR model with 6 h of ischemia followed by blood flow reperfusion, is suitable to reflect the conditions under the ischemia and IR injury that occur in this group of PAD patients. With Evans blue evaluation, we found that the blood flow was significantly restored 18 h after the reperfusion procedure (Figure 4). It is also noted that the blood flow restoration in the white muscle was more significant even when compared with the sham control. This is consistent with the tissue hyperperfusion condition reported in post-revascularization PAD patients (Beckman, Schneider, & Conte, 2021). In the observation for prolonged time points such as IR 66 h and IR 114h, the blood flow in the white portion of the gastrocnemius muscle was still persistently higher than that in the FAO 6 h. However, no significant difference was found between IR 66 h and IR114 h. This partly suggested the occurrence of new thrombolysis or ischemia following the surgery of reperfusion, which is also among the commonly seen complications in PAD patients following revascularization (Parvar et al., 2021). Therefore, this IR model is likely to reflect the state of IR injury, blood flow restoration, and features of the critical complications in PAD patients undergoing revascularization surgery. This paves the way for our present study to develop a rat model of IR with 6 h of hindlimb ischemia followed by different time points of reperfusion.

In the present study, we examined the Evans blue concentration in the skeletal muscles of the lower extremity to indicate the blood flow supply for this location. In the gastrocnemius muscle, we divided the muscle into type I slow-twitch (red) and white portion based on the site and the appearance (Winder et al., 1974; Matsakas et al., 2006). Red muscle fibers produce ATP through oxidative phosphorylation. In contrast, white muscle fibers produce ATP through aerobic or anaerobic respiration (glycolysis) to meet the energy demand during fast muscle contraction. Due to the less energy supply for long-term muscle movement, white muscle fibers are prone to fatigue much faster than the slow-twitch muscle fiber (Booth & Thomason, 1991). During muscle contraction, the EPR is partly evoked by the metabolites (e.g., ATP, lactate, and proton) through the metabo-receptors in the muscle afferent nerves ending in the muscle interstitium. The soleus muscle’s static contraction (mainly consisting of oxidative red muscle fiber) induced no increase in BP. In contrast, the contraction of the medial gastrocnemius muscle (mixed by both slow and fast-twitch fiber types) caused an increase in BP (Petrofsky & Lind, 1980). A further study reported that the static contraction of a predominately glycolytic (white) muscle evoked a more enormous pressor response as compared with that elicited by the contraction of a primarily oxidative (red) muscle (Wilson et al., 1995). Results of the present study indicate that FAO 6 h significantly reduced the blood flow in the plantar muscle, red and white portion of the gastrocnemius muscle. Following the reperfusion, the blood flow was partly recovered in the plantar and gastrocnemius muscles (Figures 4A–C). Interestingly, in the white portion, the blood flow was slightly overcompensated (∼120.3% of the sham group level at the early stage of IR 18 h (Figure 4C). The overcompensation of the blood flow can introduce more reactive oxygen species (ROS) to the perfused tissues, especially during the early stage. Along with the results in the previous studies (Chan et al., 2004; Charles et al., 2017), it is indicated that the white muscle is likely to be more vulnerable to IR injury than the red muscle. Hence, the IR injury in the white muscle likely plays a vital role in the amplified EPR response induced by IR.

The present study is also an initial report regarding the EPR response to static muscle contraction of the hindlimb in decerebrated rats with 18–114 h of blood flow reperfusion. In consistence with the previous report showing increased BP response to dynamic exercise following forelimb IR in mice, our current result demonstrated that MAP response in IR 18 h rats was exaggerated (Figure 5A). It is important to note that, compared with the global dynamic exercise, the approach of decerebration in rats entirely eliminates the regulatory effect on the BP response that arises from the cortex and regions above the mid-brain (i.e., central command). In contrast, the BP response during static muscle contraction is specifically induced by the stimulation of the Group III and IV muscle afferents embedded in the interstitial space of the skeletal muscle (Sapru & Krieger, 1978; S. A. Smith et al., 2001; Stone, Yamauchi, & Kaufman, 2014; Xing, Lu, & Li, 2013). This makes our approach a better way to study the underlying regulatory mechanisms of the BP response induced by the metabolic products from the exercising muscle of IR rats. In combination with the Evans blue experiment results, it also indicates that IR 18 h is a suitable time point for future mechanism studies regarding the IR-induced EPR response.

In a human study, a follow-up examination of the EPR response was performed in PAD patients after a month of revascularization surgery. It was found that the revascularization significantly attenuated the exaggerated EPR when compared with the pre-surgery condition. Results of this previous study suggest the promising efficacy of revascularization surgery on increased BP response in PAD patients (Miller et al., 2018). Non-etheless, the EPR response is largely unclear in PAD patients compared to age-matched healthy control participants. Using an IR rat model, our present study indicated that the exaggerated BP response was the most profound in IR 18 h and tended to decrease with the prolonged IR time. However, the exaggerated BP response remains through time points from post-IR 18 h–114 h (Figure 5A). This suggests that, although recovery of the blood flow significantly improves the EPR, IR injury is still one of the most predominant complications and may have accentuated impacts on the EPR in PAD patients following the revascularization surgery. Thus, it brings our further attention to the effects of such post-revascularization complications on PAD patients and encourages future studies on intervention strategies to improve the recovery of post-revascularization PAD patients.

## Limitation

In real-world situations, PAD patients’ activities consist of multiple dynamic forms (i.e., walking). The dynamic exercise-induced BP response is increased in a mice model with IR (Ross, Queme, Shank, Hudgins, & Jankowski, 2014). Non-etheless, in the present study, we evoked the EPR *via* static muscle contraction by specifically stimulating the Group III and IV muscle afferents. Taken together, data suggest that IR causes an exaggerated EPR during both dynamic and static exercise. Meanwhile, results of this study were obtained from adult male rats. Note that previous studies on human participants indicated that the difference in pressor response between older and younger groups depends on the range of blood flow restriction (Markel et al., 2003). The difference among the different ages of animals is necessary to be specifically examined in future studies. It should also be noted that the IR-induced EPR response is equally important to be studied in females because both human and animal studies have shown that the EPR response is lower in females than in males (Schmitt & Kaufman, 2003; J. R. Smith, Koepp, Berg, Akinsanya, & Olson, 2019). Considering that the primary scope of the present study was the effects of IR on the EPR response, we included only male animal in the present study. A study design with simply female animals and determination of the role of female sex hormones in regulating the IR-induced EPR response can better clarify an issue with respect to sex difference. Therefore, further studies, e.g., EPR response in IR and underlying mechanisms, are needed to explicitly be performed on female animals.

## Conclusion

In this rat model of hindlimb IR (Figure 1), the blood flow to the hindlimb muscle is significantly reduced following the femoral artery ligation and partially recovers after the blood flow reperfusion. Moreover, femoral artery occlusion significantly exaggerates static exercise-induced BP response in rats, and the exaggerated BP response remains through time points from post-IR 18–114 h. Investigations on the underlying molecular mechanisms are warranted. For the translational significance, attention should be paid to the clinical study of the exercise-induced BP response in post-revascularization PAD patients and appropriate and effective interventions alleviating IR injury for post-revascularization PAD patients.

## Data Availability

The original contributions presented in the study are included in the article/supplementary material, further inquiries can be directed to the corresponding authors.
